# Detection and classification of peaks in 5' cap RNA sequencing data

**DOI:** 10.1186/1471-2164-14-S5-S9

**Published:** 2013-10-16

**Authors:** Dario Strbenac, Nicola J Armstrong, Jean YH Yang

**Affiliations:** 1 School of Mathematics and Statistics, University of Sydney, NSW, Australia

## Abstract

**Background:**

The large-scale sequencing of 5' cap enriched cDNA promises to reveal the diversity of transcription initiation across entire genomes. The process of transcription is noisy, and there is often no single, exact start site. This creates the need for a fast and simple method of identifying transcription start peaks based on this type of data. Due to both biological and technical noise, many of the peaks seen are not real transcription initiation events. Classification of the observed peaks is an essential filtering step in the discovery of genuine initiation locations.

**Results:**

We develop a two-stage approach consisting of a fast and simple algorithm based on a sliding window with Poisson null distribution for detecting the genomic locations of peaks, followed by a linear support vector machine classifier to distinguish between peaks which represent the initiation of transcription and peaks that do not. Comparison of classification performance to the best existing method based on whole genome segmentation showed comparable precision and improved recall. Internal features, which are intrinsic to the data and require no further experiments, had high precision and recall rates. Addition of pooled external data or matched RNA sequencing data resulted in gains of recall with equivalent precision.

**Conclusions:**

The Poisson sliding window model is an effective and fast way of taking the peak neighbourhood into account, and finding statistically significant peaks over a range of transcript expression values. It is orders of magnitude faster than doing whole genome segmentation. The support vector classification scheme has better precision and recall than existing methods. Integrating additional datasets is shown to provide minor gains in recall, in comparison to using only the cap-sequencing data.

## Background

The locations of transcription start sites (TSSs) in the genome are of biological importance. Transcription factor binding sites (TFBS) are generally located within close proximity to annotated TSSs and are thought to regulate the packing of nucleosomes [1]. There is rarely only a single TSS for a particular transcript [2]. We refer to clusters of TSS for a single transcript as TSS regions. Nucleosome positioning determines the accessibility of the transcription start region to RNA Pol II. Knowing the locations of the TSS regions reduces the genomic regions in which to search for regulatory motifs and generate hypotheses about the cause of changes in gene expression. For example, the *Prkd2 *promoter contains a *Gabp *binding site. When there is a loss of *Gabp*, *Prkd2 *expression is much reduced, and can lead to the development of chronic myelogenous leukemia [3]. Correct usage of alternative TSSs is also important for healthy development of the nervous system [4]. This highlights the importance of transcription start detection to human health.

Cap-analysis gene expression sequencing (CAGE-seq) is a high throughput sequencing technology that provides millions of short reads per biological sample, representing the variety of transcription initiation and recapping locations in a cell type [5]. Briefly, the RNA is reverse transcribed into a single strand of cDNA. Biotin is added onto both ends of the newly synthesised molecule. Only the 5' end biotin label is captured with streptavidin on magnetic beads. The single stranded cDNA is then released and sequenced. Due to its cost, CAGE-seq has been mostly performed by the FANTOM consortium, one of whom invented the technique [6,7]. CAGE-seq is, however, becoming more widely used [8].

The CAGE reads typically represent the first thirty bases next to the 5' cap site, which is bound to the first RNA base of a transcript. The sequence of the read is mapped to a reference genome, to determine its location. Because CAGE reads are not supposed to be spliced, any general-purpose short-read mapping algorithm, such as Bowtie [9], could be used. Once the location is determined, only the first base is considered in further analyses. Even for well-characterised transcripts, there is a spread of positions which have first base signals, and need to be grouped into units of peaks.

One caveat of CAGE-seq recently discovered is that it also enriches for recapped RNA, which means that regions are detected where there is no transcription initiation [10,11]. The startling overlap between CAGE reads in exons and small RNA sequencing datasets suggested cleaving and recapping throughout many RNA molecules was occurring. Also, some CAGE reads started less than 20 bases from exon boundaries and mapped across them. In addition, a protein has been discovered that cleaves and recaps RNA in the cytoplasm [12]. This established the functional mechanism causing recapping, and provides support to the earlier interpretation of the overlap of small RNA and CAGE-seq datasets.

**Figure 1:**
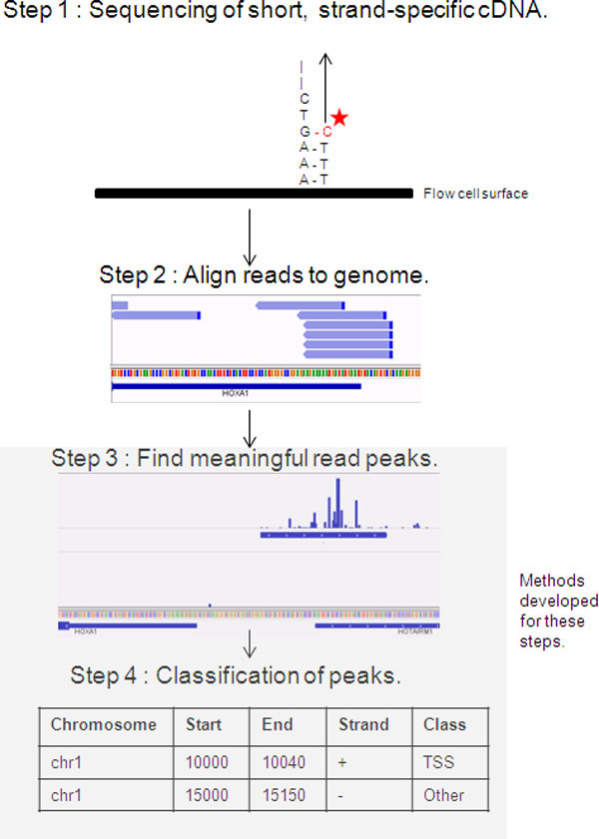
**Key steps in the bioinformatic workflow for analysing CAGE sequencing data**. The reads from the sequencer are aligned to the genome. Only the first position of each read is used, and the positions are clustered into peaks. Lastly, a classification algorithm needs to be used to label the peaks as being from transcription initiation or not.

To date, a small number of methods are available to analyse CAGE-seq data. The general workflow consists of mapping the reads, creating positional histograms of read start position counts, finding statistical differences in read density along a sequence (peaks), and determining whether the identified peaks are TSS or not (Figure 1). With the exception of a single method [13], peak classification is always ignored [2,6,14-16].

CAGE-seq analysis is currently in its infancy, and algorithms proposed previously do not provide results that are required by biologists. Our two-stage approach provides a solution to both critical steps of analysis. Unlike previous methods, the peak finding algorithm is fast and provides visually intuitive peaks. Our SVM-based classification gives high precision and recall values, and compared to both Segway and the ENCODE HMM method, is currently the best performing classifier for CAGE-seq data. Investigation of the benefits of integrating external data sources into the classification allows us to make informed recommendations. Pooled external data from a public database is simple to obtain at no cost, but is not representative of the cells studied by CAGE-seq. Generating matched RNA-seq data is time and money consuming, but is specific to the cell type under study. Pooled external data was just as beneficial as integrating matched RNA-seq data, suggesting that the extra effort of RNA-seq provides no significant benefit.

### Previous approaches

Various existing methods are available for the task of calling peaks. The first method for calling peaks in CAGE-seq data groups reads into clusters if they overlap by at least one base [14]. This is likely to join positions that are thousands of bases away for highly expressed transcripts. It also lacks any measure of statistical significance. A more recent approach using the Maximal Scoring Subsequences algorithm [2], implemented in the software package Paraclu, relies on exhaustively using all possible values of a penalty parameter that is used to define the breakpoints of peaks. The sheer number of results it returns, many of which overlap multiple genes, means it requires manual post-processing to arrive at a sensible number of peaks, which have biologically meaningful widths. A third approach is based on looking for adjoining positions with CAGE reads that have constant relative expression across multiple samples [15]. However, the recapping signal near TSS peaks also has constant relative expression at nearby positions, and this algorithm often generates peaks that are too wide to be biologically meaningful [P. Balwierz, pers. comm.].

The results of the peak calling algorithms above depend on read density, and do not classify peaks as originating from TSS or otherwise. The only algorithm specifically designed to classify CAGE peaks is based on modelling k-mer frequencies surrounding the peaks using an unsupervised hidden Markov model [13], herein called the ENCODE HMM method. The k-mers used in training are weighted proportionally to the number of reads in a peak. In other words, the algorithm biases towards learning the features of CAGE peaks with high read counts, and against peaks for lowly expressed genes. Strangely, no validation of results from the classifier is performed in the original article, and the results were used as if they were all correct. The authors also did not consider integrating external data in their model, which could potentially improve the algorithm performance. The simplest approach that avoids peak classification (and peak finding) altogether is to make small counting windows around annotated TSS [16], before performing an analysis of the amount of signal. The drawback is that novel transcription starts, and even novel genes, are ignored.

## Methods

### Datasets and preprocessing

Peak finding was performed on publically available CAGE-seq data. Features used for feature selection and classifier development were also obtained online. Classification results of two existing methods were obtained online.

#### ENCODE project repository

CAGE data was obtained for six cell lines (GM12878, H1-hESC, K562, HeLa-S3, HepG2, and HUVEC - the CAGE cell lines) by downloading the mapped BAM files from the ENCODE data repository [17] on the UCSC Genome Browser website. Preprocessing details are found elsewhere [13]. The unique Submission IDs are 3946, 2380, 2359, 2363, 2381, and 2376.

Unmapped, total RNA-seq data for two of the six CAGE cell lines (GM12878 and K562) was downloaded. Total RNA-seq data is not available for the other four cell lines. The unique Submission IDs are 1502 and 1503. Quality control of the downloaded files indicated that they are likely not from a single end sequencing experiment (Additional file 1). The dip in quality before the middle of the horizontal axis and the fact that it was technically impossible to generate 152 base reads in 2009, suggests two paired end read files were merged into a single text file. Data cleaning involved splitting the reads down the middle, recreating the read IDs with correct pairing information, and writing two separate files of reads. Raw reads were mapped to the human genome assembly hg19 with STAR version 2.3.1c [18]. Non-default options set were --outFilterMultimapNmax 1 --outFilterMismatchNmax 3 --clip3pNbases 40 --alignIntronMax 100000. Only uniquely mapping reads and no more than 3 mismatches to the reference sequence were allowed. 40 bases from the ends of each pair of reads were ignored. No splice junctions spanning more than 100000 bases were allowed.

Pooled measurements of transcription factor binding from 95 cell types of an unspecified number of transcription factors stored in the table wgEncodeRegTfbsClusteredV2 were downloaded. Pooled DNAse I hypersensitivity data using 74 cell lines was obtained from the table named wgEncodeRegDnaseClustered. H3K4me3 data was not integrated by ENCODE, so seven standardised signal files were downloaded, with Submission IDs 2806, 2815, 2846, 2878, 2890, 2909, and 2921. Four of the cell lines are CAGE cell lines.

The peaks classified as TSS by the ENCODE HMM were downloaded with Submission IDs 5610 and 5147. Five of the cell lines have the same submission identifier, although the classification results were all confirmed to be sample-specific.

#### UCSC table browser

The phyloP46wayPlacental track of conservation between 46 mammalian genomes was downloaded. Scores indicate the rate of evolution. Higher scores represent bases that are evolving faster and less conserved than the null hypothesis of neutral evolution.

#### FTP directories

Segway segmentations of the CAGE cell lines [19] were obtained from an URL provided to us by the authors of Segway (http://ftp.ebi.ac.uk/pub/databases/ensembl/encode/awgHub/byDataType/segmentations/jan2011/).

#### GENCODE genes

The file gencode.v15.annotation.gtf.gz containing the latest transcript annotation of the human genome, version 15, was downloaded from the GENCODE data portal [20].

### Peak finding

For a method to work generally for both high and low peaks, the null distribution of the statistical test should be different for each candidate region. Our method is similar in spirit to the popular ChIP-seq peak finding method MACS [21], but tailored to the particular characteristics of CAGE-seq data. CAGE reads with mapping quality of less than 20 are discarded. The definition of short read mapping quality is described elsewhere [22]. Only the first position of a CAGE read is used in the analyses due to the fact that it is potentially representative of the site where transcription started. A candidate window of width *w *is moved along each strand of each chromosome in increments of w/2. Based on biological intuition about the range of widths of peaks overlapping known transcription starts, w=50 is used. Also, flanks on either side of the candidate window are made. The width of both of these windows was chosen to be 200. For each candidate window, and its two flanking windows, counts of CAGE read starts are made. Any candidate window with less than 10 reads is immediately discarded. The counts in the flanks are scaled for their window sizes relative to the candidate window; in this case, dividing by 4, and rounded to the nearest integer. The counts are assumed to be Poisson distributed, and the probability of observing a count as high, or higher than the candidate region is calculated twice, with  λ equal to one of the scaled flank counts each time. If in either statistical test the probability is below $10^{-20}$, then the candidate window is added to a list of peak windows. The ends of peak windows are trimmed for outermost, contiguous positions that contain zero counts. Finally, any peak windows separated by less than 30 base pairs are merged into a single peak.

### Feature construction

In total, eight features were constructed, as described below.

#### Kurtosis

Pearson's kurtosis, based on the fourth standardised moment, is used. This feature is included to examine if any differences in peak shape would be discriminatory.

#### Read density

The number of CAGE reads inside the boundaries of a peak, divided by the width of a peak.

#### Mammalian conservation

Considered for its potential correlation with regulatory regions, such as promoters, scores inside the peaks were used. For each peak, the single base conservation values were averaged. A small fraction of peaks did not overlap with any bases with conservation scores, because the genomic sequence was not able to be multiply aligned to the other genomes. For these peaks, we used an imputed value equal to the minimum value of peaks that had conservation scores.

#### TFBS

Often enriched near locations where transcription starts. For each peak, the maximum score for each feature in a window extending 100 base pairs from the peak ends was assigned to the peak. The measured maximum is used so as to be permissive, rather than exclude cell type specific signals.

#### DNAse I hypersensitivity

Considered as TSSs typically occur in open chromatin. Similar to TFBS, the maximum count within 100 base pairs from selected peaks are determined.

#### H3K4me3

This histone modification is known to be found on the nucleosomes surrounding active TSSs. Again, we used the maximum score within 100 base pairs, as for TFBS, and DNAse I hypersensitivity.

#### 4-mers counts

Patterns of DNA bases surrounding the TSSs are also known to be different to other regions in the genome [23]. A 500 base pair window was created upstream and another downstream of the summit of each CAGE peak. Frequencies of all 4-mers were calculated independently for the two windows. In the upstream window, there are 4^4 ^= 256 distinct 4-mers, and similarly downstream, making a total of 512 4-mer features.

#### RNA-seq difference

The number of RNA-seq reads on either side of the peak was counted. Since there would be numerous reads expected downstream of a TSS region and none immediately upstream, two counts were made. One count is a 100 base wide flanking window immediately upstream of the 5' edge of the CAGE peak. The other is the same size, but downstream of the 3' edge of the peak. The feature calculated was P(Y≤y) of the Poisson distribution where  λ is equal to the downstream flank count and *y *is the count in the upstream flank.

Table 1 provides a summary of all features and their calculation. PCA was used to determine which of the calculated features would be included in the classification analyses. Principal components were calculated and the first two dimensions visualised. Features that had $\left|\rho \right|\geq 0.5$ with components that appeared to separate samples by class labels were retained. Correlation was calculated as the component loading multiplied by its eigenvalue. This procedure was done separately for the single features and the 4-mer multiple feature. The combinatorial number of 4-mer features means that they add a large amount of total variance without necessarily being informative, which PC1 will explain. Selected features were standardised to be between 0 and 1 by dividing by the maximum score for all peaks, per feature type and per cell line.

**Table 1:** Number of peaks found by Poisson thresholding of sliding window method.

Name	Summarisation	Location	Type
Kurtosis	Directly used	Peak	Internal
Read Density	Directly used	Peak	Internal
4-mers Counts	Count	500 bases upstream and downstream of peak summit	Internal
TFBS	Maximum	Peak and 100 base extension of boundaries	External
DNAse I Hypersensitivity	Maximum	Peak and 100 base extension of boundaries	External
H3K4me3 Hypersensitivity	Maximum	Peak and 100 base extension of boundaries	External
Mammalian Conservation	Average	Peak	External
RNA-seq Difference	Distribution function probability	100 bases flanks adjacent to peak boundaries	External

For each feature, the summarisation procedure, location of data points summarised, and the feature categorisation are shown.

### Peak classification

Class labelling of peaks was made by the same method used for Segway [19]; Segway is, to date, the most comprehensive study of TSS region determination. Unlike typical classification datasets, where the true class membership is clear and known in advance, TSS datasets require the assignment of inferred class labels to peaks. Briefly, 500 base windows were made upstream and downstream of the start position of each GENCODE transcript. If a CAGE peak overlapped with any of the windows, it was labelled as a TSS peak. Otherwise, it was assigned to the non-TSS group.

SVM training was done with a L2-regularised L2-loss linear SVM and the primal solving option was chosen. This is because there are as many variables to optimise as there are features in the primal form, and there are many more CAGE peaks than peak features. A broad range of cost values was examined, to understand classification performance at different hardness levels of the SVM margin. To handle imbalanced class sizes, error weights were provided for each class. Not adjusting for differences in set sizes of the smaller TSS and larger non-TSS set would result in high accuracy for the non-TSS set and low accuracy for the TSS set, since the default parameterisation of SVMs is to maximise the overall number of correct predictions [24]. For the TSS class, the weight was calculated as the number of peaks in the non-TSS class divided by the number of peaks belonging to the TSS class. For the non-TSS class, the weight was 1.

Performance of the classification was evaluated by precision and recall. Precision is the percentage of TSS classified peaks that are labelled as TSS peaks. Recall is the percentage of labelled TSS peaks that were classified as TSS peaks. Leave-one-out cross validation (LOOCV) was used with five cell lines for training and one for testing, in each round. Precision and recall values were compared to those of Segway and ENCODE HMM, to determine which method performs best for TSS region prediction, overall.

### Computing environment

All analyses were performed in R [25] version 2.15.3. Packages from the Bioconductor [26] project were used extensively. Mapped data was read into R by using the Rsamtools package. The package GenomicRanges was used for overlapping genomic intervals and creating flanking regions. The function oligonucleotideFrequency from Biostrings was used for calculating all 4-mers based on the sequence of the hg19 genome found in the package BSgenome.Hsapiens.UCSC.hg19. The rtracklayer package was used for importing ENCODE feature tracks and exporting coverage and peak region tracks. The CRAN package "moments" was used to calculate peak kurtosis. SVM training and prediction were performed with the R interface to LIBLINEAR [27], LiblineaR.

## Results

### Peak finding

**Figure 2:**
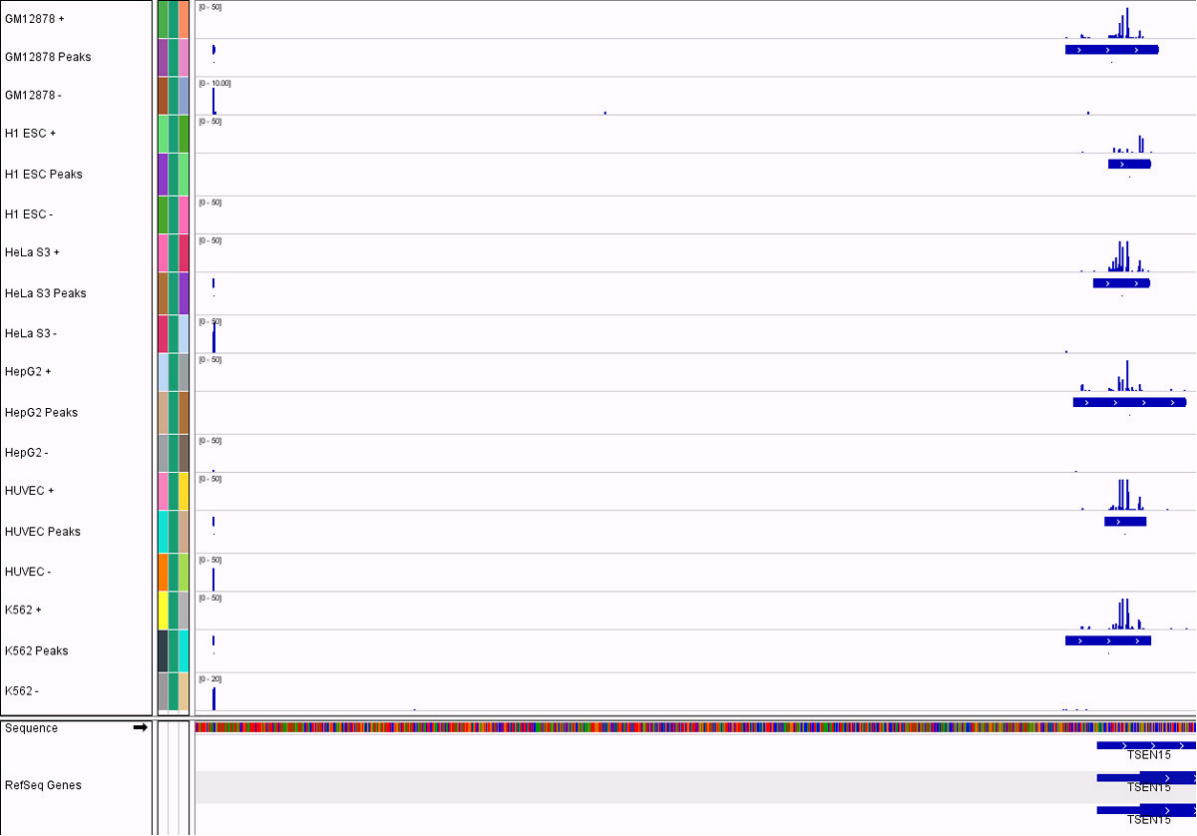
**Peaks found by the algorithm in six cell lines**. CAGE-seq strand-specific read coverage is shown along with tracks that contain boxes representing the areas that are found to be peaks. A narrow peak of between 1 and 3 bases wide is at the left side of the figure. A wider peak that varies between 76 bases and 207 bases is located at the right side.

The local Poisson thresholding algorithm discovered tens of thousands of peaks in each sample (Table 2). About twice as many peaks were found for the H1-hESC cell line compared to any other cell line. This is biologically expected, because stem cells have open chromatin and transcription of many tissue-specific transcripts occurs, which are otherwise silenced in differentiated cells [28]. Manual exploration of coverage tracks showed that the algorithm finds peaks both broad and narrow (Figure 2). By definition, the algorithm will not find extremely broad peaks that were rarely observed, some of which are thousands of bases wide. However, based on current biological knowledge, these peaks are not likely to be real TSS regions, and were observed to overlap with known long 3' UTRs.

**Table 2:** Summary of features and how they are calculated.

Cell Line	Total Peaks Detected
GM12878	43161
H1-ESC	111945
HeLa-S3	41195
HepG2	59390
HUVEC	40420
K562	35622

For the six ENCODE cell lines used, the total number of peaks found by the sliding window approach is shown.

### Feature selection

**Figure 3:**
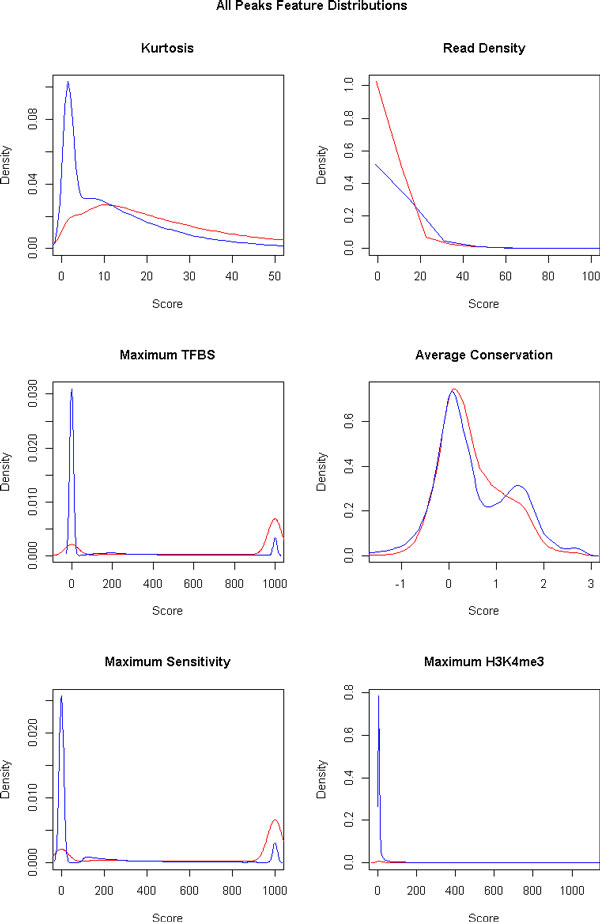
**Density plots of single feature scores for all peaks in all cell lines**. Red line is for TSS class. Blue line is for non-TSS class. Kurtosis and read density are internal features. The other four features are external features.

Feature selection is an important step in any classification algorithm, as features not correlated to the class distinction can adversely affect the prediction performance. All peaks from all cell lines were used in this step, so as to ensure features selected are those which generalise well. Initial exploration of the association of single features with classes suggested that some features would be better discriminators than others (Figure 3). Higher scores were generally observed for the three pooled external features and kurtosis, for TSS class peaks. The three pooled features are known to be positively correlated with TSS regions. The relationship of higher kurtosis for the TSS peaks than non-TSS peaks is expected, as TSS regions are known to be taller and narrower than non-TSS regions [2]. These observations motivate the use of feature selection.

Single feature selection was done based on standardised scores, as described in Methods, for all six single features. The first dimension had good separation, and largely corresponded to the separation between samples of the two classes. Those with an absolute correlation to PC1 of more than 0.5 were selected to be in the model. The features selected were: kurtosis (ρ=-0.53), transcription factor maximum (ρ=-0.92), DNase I hypersensitivity maximum (ρ=-0.91), and H3K4me3 maximum (ρ=-0.86). These are the same features that were observed to be different between classes in the density plots.

In addition to the single features, we also examined the multiple feature of 4-mer frequency. PCA was used on standardised counts of the 512 distinct 4-mers. The first principal component had good separation of the two classes. There were 168 4-mers that had |ρ| ≥ 0.5 with PC1, and were selected to be used in classification.

Among the selected features, we can broadly categorise them into internal and external features. Firstly, internal features have the characteristic of being directly computable from the mapped CAGE-seq data. Kurtosis and 4-mer frequencies are the internal features. The other features are external features. They must either be obtained from external databases or experimentally derived. In the next section, we examine classifier performance in a range of different feature scenarios.

### Classification evaluation

**Figure 4:**
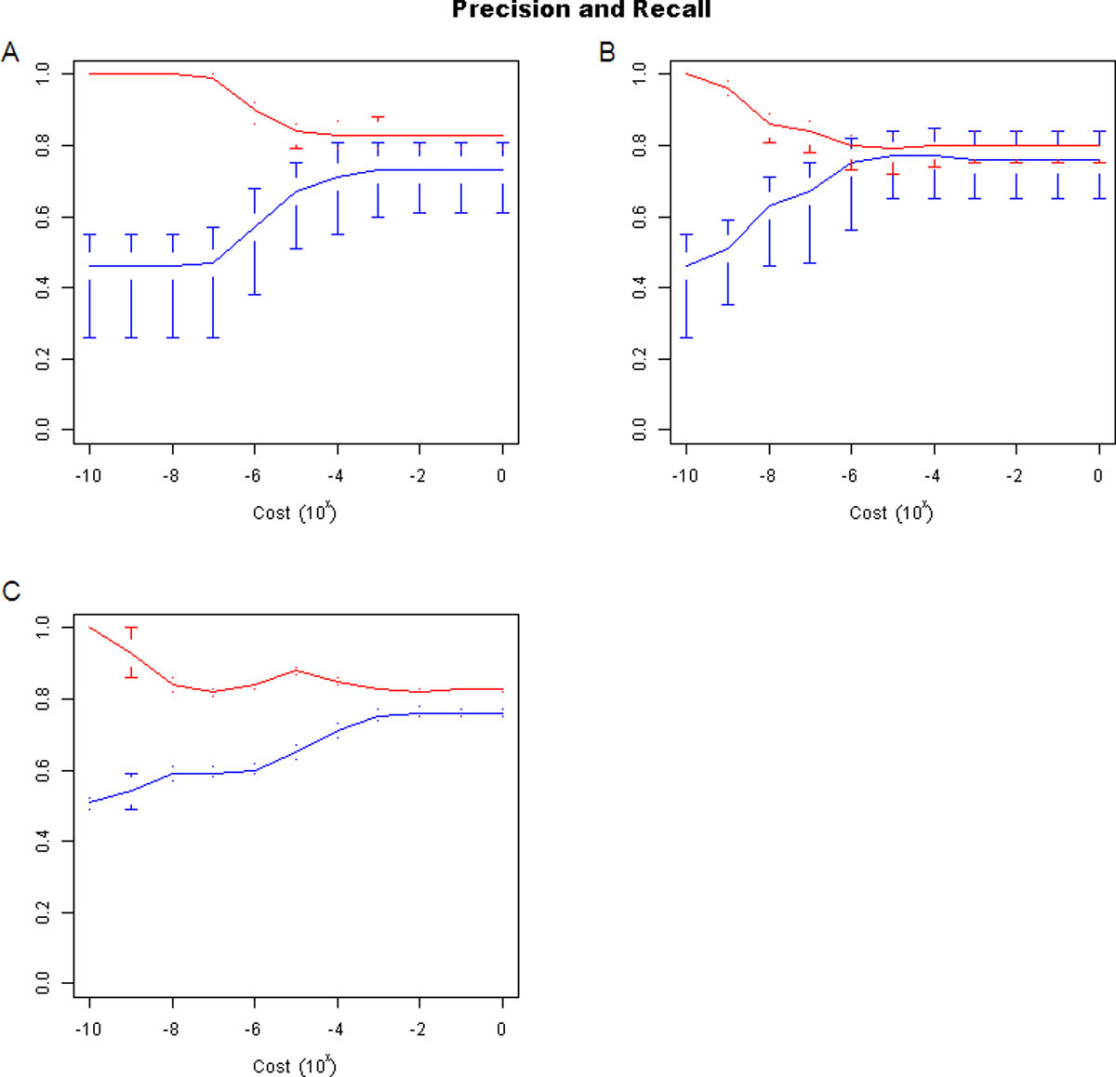
**Precision and recall for three feature scenarios**. Precision and recall are calculated at each cost parameter value based on a LOOCV scheme. Blue lines are precision. Red lines are recall. Horizontal bars or dots represent the minimum and maximum value of all cell lines. Points on the line are averages across all cell lines. A. Internal features for six cell lines. B. Internal features and pooled external features for six cell lines. C. Internal features and matched RNA-seq data for two cell lines.

We used a linear SVM to classify peaks based on the selected features. Several different cost parameters were investigated. At each level, LOOCV was performed. In the first scenario, only internal features were considered. These are kurtosis and 4-mer counts. Kurtosis and each 4-mer were initially scaled to be in the range [0,1]. To combine the kurtosis and 168 different 4-mers so that they have equal weighting in the classifier, the values of kurtosis were rescaled to be in the range [0,168]. Figure 4A shows the precision and recall values for this SVM across a large range of cost values. Precision and recall are high for most of the cost parameter values.

In the second scenario, the internal features were combined with the unmatched external features. Three external features were selected previously - namely TFBS, DNAse I hypersensitivity, and H3K4me3. They are rescaled to each be in the range [0,2×168/3], so that the contribution of all three external features is the same as the set of internal features. Figure 4B shows precision and recall values for this feature set. Precision is essentially the same as for the internal feature set, while a moderate improvement of recall is observed. Genome browser tracks of peak locations for this scenario are available (Additional File 2).

Finally, the matched RNA-seq dataset was considered, because it is desirable to determine if integration of a complementary RNA-seq experiment with internal features can improve peak classification. The RNA-seq feature is scaled to have equal importance as the internal features by setting its range to be $\left[0,2\times 168\right]$. Figure 4C shows precision and recall values for this feature set. Recall is better, relative to the internal feature set.

Performance comparisons were made to Segway and ENCODE HMM, to determine the best currently available method for TSS determination. The precision and recall of Segway (Table 3) was calculated for all six cell lines, using the same definition as in the publication, but with a current genome annotation. Segway's precision is comparable to our method across all feature scenarios. Recall, however, averages 71 % for Segway, whereas it averages 82 % for our method when considering cost parameters $\geq 10^{-4}$. Evaluation of the ENCODE HMM was also performed. The reference labelling was generated in the same way as for Segway. Precision and recall results of ENCODE HMM are also presented in Table 3. As expected from its assumptions, the algorithm has good recall (mean 0.92) at the expense of precision (mean 0.27). Our proposed SVM-based method has much better mean precision than ENCODE HMM.

**Table 3:** Precision and recall of publically available classifications.

	Segway		ENCODE HMM		Proposed Method	
**Cell Line**	**Precision**	**Recall**	**Precision**	**Recall**	**Precision**	**Recall**

GM12878	0.7	0.64	0.25	0.92	0.77	0.81
H1-ESC	0.59	0.71	0.27	0.89	0.61	0.81
HeLa-S3	0.79	0.66	0.32	0.91	0.76	0.87
HepG2	0.59	0.59	0.23	0.93	0.69	0.79
HUVEC	0.82	0.67	0.26	0.94	0.81	0.85
K562	0.77	0.62	0.27	0.93	0.71	0.88

The reference labelling is the collection of segments that overlap a GENCODE transcript with at least 2 supporting CAGE reads within 500 bases either side of the annotated start location. An example of the proposed method's performance is presented alongside the two public methods, for a SVM cost parameter value of 0.1 and using only internal features.

## Discussion

We propose a two-stage approach for the identification of TSS sites in CAGE-seq data. The first stage involves a novel algorithm to determine the peaks of CAGE reads across the genome. This method utilizes a sliding window approach with peak calling based on a local Poisson threshold that allows us to automatically detect a large number of visually meaningful peaks. In the second stage, we build a classification framework to determine which of these peaks are representative of real transcription initiation. This is achieved through employing a collection of internal and external features together in a linear SVM classifier.

A good evaluation framework for peak finding methods in CAGE-seq data is still a challenging task. Although the peak locations found by the sliding window algorithm appear to be intuitively correct, there is no objective quality metric that could be used to fairly compare it to other peak-finding methods. In other fields, like transcription factor binding site sequencing, many peak finding algorithms don't provide any measurement of peak quality [29,30]. Sometimes, a surrogate measure of how well the algorithm performs is the percentage of peaks which contain the transcription factor's binding motif [31-33]. However, it is only possible to calculate this measure if the transcription factor has a known binding motif. In addition, it is impossible to assess false negatives, because a provably complete and correct experimental method does not exist. The general field of peak finding in high throughput sequencing data would benefit greatly if it were possible to generate a truth set to compare algorithm performance.

Our study shows that improvement by integrating RNA-seq data isn't as evident as expected; the classification model with simple features shows comparable performance to the more feature-rich models. We examined three classes of features here: internal features, external (non-matched) features and external cell-specific features (RNA-seq). Internal features performed well, in terms of both precision and recall. Adding pooled external data for DNA accessibility, transcription factor site density, and an epigenetic modification known to be associated with TSS peaks resulted in a minor improvement in identifying true TSS peaks. Likewise, adding matched RNA-seq information to the internal feature set for two of the cell lines did not noticeably improve precision, while recall improved.

Even with the myriad of 'omics data, identification of TSS regions remains a non-trivial task. The ENCODE HMM algorithm is dominated by false positives. This is a common type of analysis bias in most of the ENCODE consortium's methods [34]. Currently, the segmentation of the genome using multiple epigenetic features appears to be the most sophisticated way to find regions of transcription initiation. In the published example using Segway, 31 different sources of data were required [19] and this complexity translates into days of training on a computing cluster [W. S. Noble, pers. comm.]. In contrast, our proposed method runs in the order of seconds on a desktop computer, and was shown to have similar precision, but noticeably better recall. The reduced computational runtime is a major advantage of our method.

The current wealth of generated CAGE data needs to be mined for biological insights, from both the ENCODE [13] and FANTOM [6,7] consortium, and would greatly benefit from the proposed SVM classification method. Conceivably, a sample preparation improvement may be developed in the future that is able to separate the recapped RNA from genuinely transcribed 5' ends, and may diminish the importance of the classification stage of our approach. However, any newly discovered knowledge relating to the recapping position can easily be incorporated into our SVM framework as additional features, enabling more accurate analysis of the many existing datasets.

## Conclusions

A two-stage approach involving a sliding window using a Poisson-based cut-off together with a SVM classifier is a simple and effective approach to computationally define TSS peaks. An evaluation study considering three types of feature sets (internal, pooled external, and matched RNA-seq) showed that the precision was comparable to Segway and recall was consistently better across each of the three training feature scenarios, even though our method runs many times faster than Segway. There are currently no other algorithms that could be applied to the classification problem with good precision and recall, and desirable run time.

## Authors' contributions

DS developed the method, implemented the algorithm and drafted the manuscript. NJA and JYHY participated in all aspects of the study and helped to draft the manuscript. All authors read and approve of the final manuscript.

## Competing interests

The authors declare that they have no competing interest.

## Supplementary Material

Additional file 1 **Plot of raw total RNA-seq data quality scores from ENCODE**. Quality scores drop in the middle of the read, then again at the end of the read, suggesting that the data is unlikely to have been generated by a single-end sequencing protocol. The vertical axis is Phred quality score. A. Cell line GM12878 B. Cell line K562. Click here for file

Additional file 2 **Archive of all peak calls**. Archive contains one BED file for each cell line. BED files can be viewed in any genome browser. Click here for file

## Declarations

Publication of this article was funded by the Australian Research Council grant DP130100488.

This article has been published as part of *BMC Genomics *Volume 14 Supplement 5, 2013: Twelfth International Conference on Bioinformatics (InCoB2013): Computational biology. The full contents of the supplement are available online at http://www.biomedcentral.com/bmcgenomics/supplements/14/S5.
