# Correction: Detection and classification of peaks in 5' cap RNA sequencing data

**DOI:** 10.1186/1471-2164-14-767

**Published:** 2013-11-18

**Authors:** Dario Strbenac, Nicola J Armstrong, Jean YH Yang

**Affiliations:** 1 School of Mathematics and Statistics, University of Sydney, Sydney, NSW, Australia

## Correction

After the publication of this work [1], we became aware of several errors.

1. The headings of Table 1 and Table 2 have been swapped. Table 1 heading should read: “Summary of features and how they are calculated” while Table 2 heading should read: “Number of peaks found by Poisson thresholding of sliding window method.”

2. In Methods section, the last sentence of ‘Peak finding’ should read: “Finally, any peak windows separated by less than **50** (not 30) base pairs are merged into a single peak.”

3. In Results section, ‘Classification evaluation’, description of Figure 4B should read: “**Recall** is essentially the same as for the internal feature set, while a moderate improvement of **precision** is observed”. Description of Figure 4C should read: “**Precision** is better, relative to the internal feature set.”

4. One of the software packages we used, Genomic Ranges has been published recently, and can now be referenced [2].

The changes do not affect the correctness of the method described. We regret any inconvenience that these errors might have caused.
