# Supplementary figures and images for: Detection and classification of peaks in 5' cap RNA sequencing data
Source: BMC Genomics. 2013 Oct 16;14(Suppl 5):S9. doi: 10.1186/1471-2164-14-S5-S9 (PMC3852351; doi:10.1186/1471-2164-14-S5-S9)

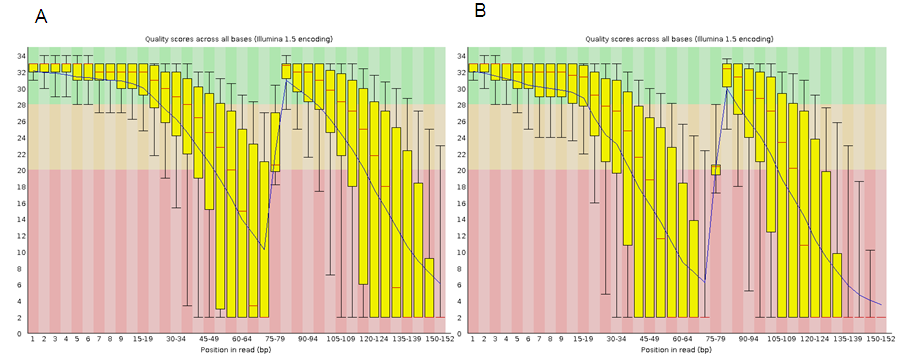

Supplement: Additional file 1 — Plot of raw total RNA-seq data quality scores from ENCODE. Quality scores drop in the middle of the read, then again at the end of the read, suggesting that the data is unlikely to have been generated by a single-end sequencing protocol. The vertical axis is Phred quality score. A. Cell line GM12878 B. Cell line K562. [file 1471-2164-14-S5-S9-S1.png]
